# First-principles framework for bifacial dye-sensitized solar cells: electronic structure, optical properties and functional dependence

**DOI:** 10.1039/d6ra05493e

**Published:** 2026-07-28

**Authors:** D. Gemeri, M. B. Milosavljević, Ž. S. Maršić

**Affiliations:** a Faculty of Science, University of Split Ruđera Boškovića 33 Split 21000 Croatia dgemeri@pmfst.hr zsm@pmfst.hr; b Center of Excellence for Science and Technology-Integration of Mediterranean Region (STIM), Faculty of Split, University of Split Ruđera Boškovića 33 Split 21000 Croatia margarita@stim.unist.hr

## Abstract

The bifacial dye@TiO_2_ architectures have recently emerged as a promising strategy to enhance light harvesting in dye-sensitized systems. However, their fundamental electronic and optical properties remain largely unexplored from a theoretical perspective. Despite growing experimental interest, a comprehensive first-principles understanding of bifacial dye-semiconductor interfaces is still missing. In this work, a systematic density functional theory (DFT) and linear-response time-dependent density functional theory (LR-TDDFT) investigation of monofacial and bifacial dye@TiO_2_ systems is presented for three representative organic dyes. The impact of bifacial functionalization on electronic structure, excitation pathways, and directional optical response is analyzed in detail. The results reveal that bifaciality is not an intrinsic property of all dyes, but instead depends critically on the spatial distribution of frontier molecular orbitals and on the exchange treatment within the chosen functional. This study provides the first consistent theoretical framework for interpreting bifacial effects in the dye-sensitized architectures and offers design principles for future bifacial dye systems.

## Abstract 2

The bifacial dye@TiO_2_ architectures have recently emerged as a promising strategy to enhance light harvesting in dye-sensitized systems.

## Graphical abstract

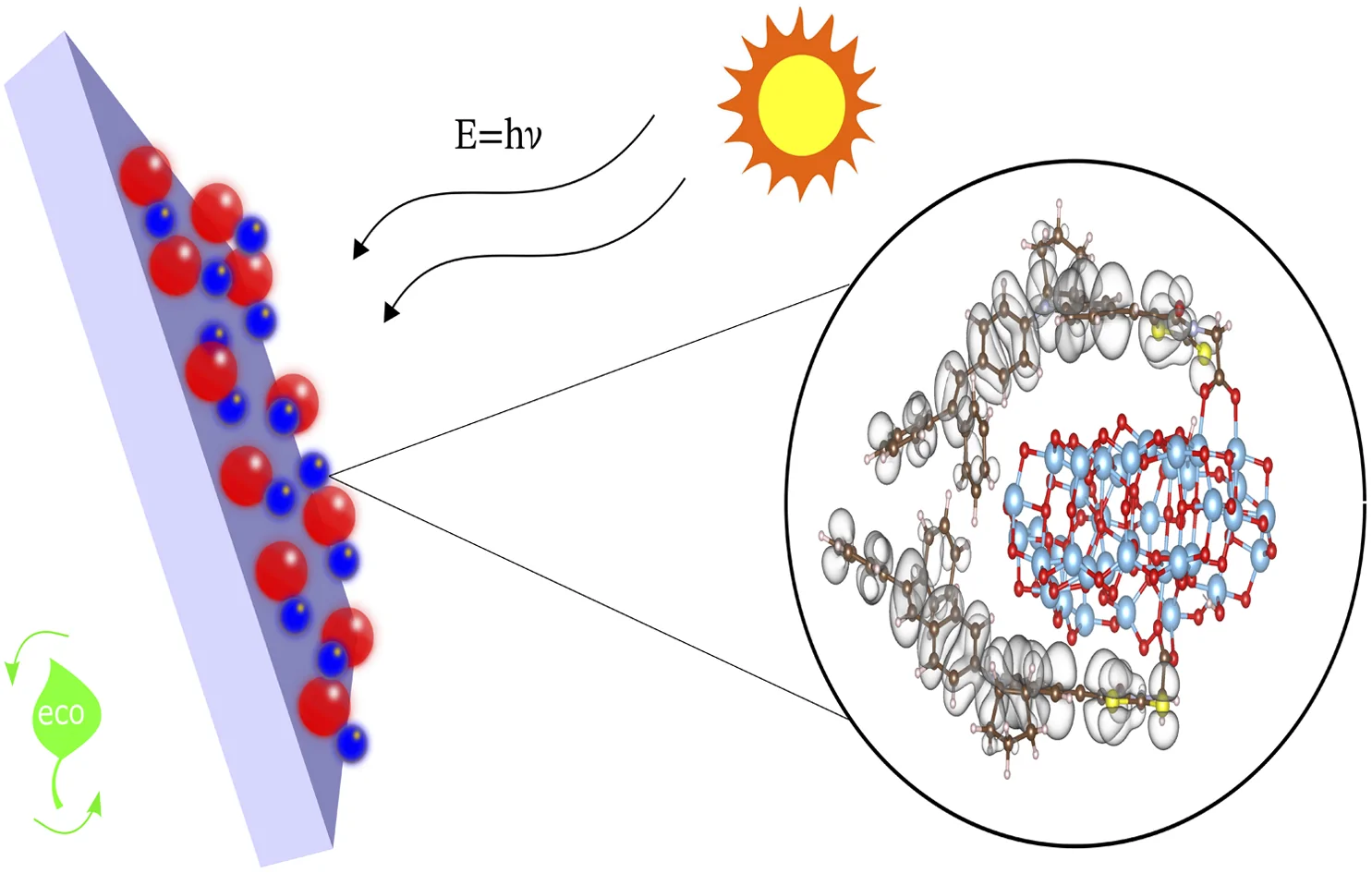

## 1. Introduction

As global energy demand is increasing every day, the quest for efficient, sustainable, cheap and eco-friendly renewable energy resources still represents an ongoing and challenging issue.^1^ It is already known that proper absorption of just a tiny amount of sunlight is sufficient to satisfy the global energy needs. The third generation of photovoltaic (PV) devices, such as dye-sensitized solar cells (DSSCs), represents one of the possible solutions.^2^ In general, DSSCs consist of three parts: a working electrode which is a mesoporous semiconductor upon which the dye is anchored, an electrolyte with a redox couple, and a catalyst layer that serves as the counter electrode. The working principle is pretty simple: photons are absorbed by the working electrode, triggering the generation of electron–hole pairs within the dye molecule. Excited electrons travel to the conduction band (CB) of the semiconductor, all the way to the counter electrode, where the circle is closed. Regeneration of the oxidized dye is done by the electron transfer from the electrolyte.^3^ DSSCs are easily fabricated from inexpensive and non-toxic materials and are currently one of the frontrunners for indoor and outdoor applications.^4^ To be fully commercialized, we need to find a way to increase the maximum power conversion efficiency (PCE) and go beyond 20% using only organic dyes.^5–8^ Thus, special attention needs to be drawn to the light harvesting and dye usage.

An idea for a solution that can possibly improve solar cell efficiency without a significant increase in production costs came from Hiroshi in 1966, when bifacial concept of a solar cell was introduced.^9,10^ Results were promising and showed some higher power production. However, only in 2009 did the bifacial approach gain some more interest, especially in the industry where they started to manufacture bifacial components based on silicon.^11^ Aside from that, we can safely say that the concept of bifacial DSSCs has barely been investigated through all these years. While some experimental papers can be found in the literature, theoretical and first-principle calculations hardly exist, as per our knowledge.^12–16^ Main goal for the bifacial solar cell is to absorb photons from both the front and rear sides of the cell, which allows more utilization of the reflected light and radiation from the surroundings (the albedo's effect), that should result in a greater generation of photoexcited electrons and overall PCE at the end.^17^ The operating principle remains the same as that of monofacial structures.

Thus, in this work, we are using density functional theory (DFT) and its extension, linear-response time-dependent DFT (LR-TDDFT), and investigate electronic structure, optical properties and density functional approximation dependence on three different dyes (WD8, D102, JK2) anchored on a semiconductor (TiO_2_), from both the front and the rear sides.^18–20^ We already used similar approach in our previous work, but solely with the monofacial counterpart, including graphene quantum dots (GQDs) and electron dynamics simulations.^21–23^ The conclusions regarding bifaciality are therefore discussed within the scope of the representative D102, JK2 and WD8 dyes adsorbed on the validated (TiO_2_)_38_ cluster model considered in this study. Indeed, we continuously compare our results for the mono- and bi-facial interfaces, using the same methods.

## 2. Theory

Density functional theory and its linear-response time-dependent density functional theory represent the most valuable and efficient methods of choice in order to explain the most important properties of the individual dyes and complex dye/semiconductor systems. On the other hand, we are familiar with the shortcomings of these methods, especially when it comes to the description of excited state properties and energy level alignments.^24–27^ For this reason, we used three different exchange–correlation functionals (global, range-separated and local hybrid functionals) to understand their possible accuracy and differences between them.

Global hybrid functionals were introduced by Becke and improved the application perspective of DFT when it comes to molecules and materials.^28^ Becke added a constant fraction of the exact exchange from the Hartree–Fock approximation which completely changed the perception, allowing for accurate prediction of band gaps and chemical properties. The general formulation is given as follows:

1 *E*^GH^_x_ = *a*_0_*E*^ex^_x_ + (1 − *a*_0_)*E*^sl^_x_.

where *E*^ex^_x_ is the exact exchange energy, *E*^sl^_x_ is a semi-local approximation, and *a*_0_ represents a fixed fraction of the exact exchange energy in the system. While we can use global hybrids to “soften” the self-interaction error (SIE) and achieve better description of static electronic correlations, we only deal with the global fraction of exact exchange, *a*_0_, which is in principle highly system dependent. That is why a compromise between the dye and the semiconductor needs to be present through the optimal value of the *a*_0_.

Quest for the more flexible approach in respect to the fraction of the exact exchange leads us to range-separated hybrid functionals.^29^ Here, we have a smart division of the Coulomb interaction between the electrons into short- and long-range parts2
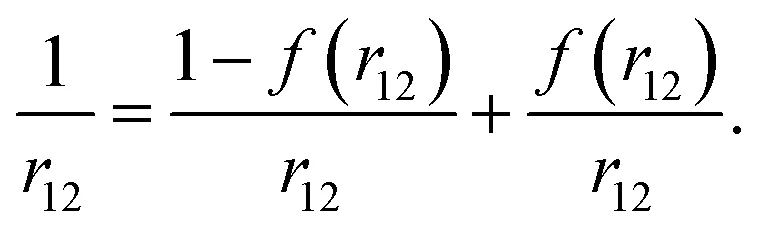
in which *f*(*r*_12_) is called the screening function and it is in charge of the smooth transition and control between short- and long-range domains. Range-separated functionals provide better description of vertical excitations, especially at the long-range, which corrects the asymptotic behaviour and is a well used method for the UV-vis spectra of some larger push–pull dye sensitizers. However, we have already showed one large limitation which has been reflected in unphysical energy level alignments when we have these type of complex dye/semiconductor interfaces.^21,22^

More recent development of local hybrid functionals proved to offer a more stable balance between global and range-separated approach.^30^

3 *E*^LH^_x_ = ∫*a*(*r*) *∈*^ex^_x_(*r*)d*r* + ∫(1 − *a*(*r*))*ρ*(*r*)*∈*^sl^_x_(*r*)d*r*.

Mixture of the exact exchange is achieved here by the local mixing function (LMF), *a*(*r*). This is a position dependent function and it governs how much of the exact exchange will be present at some specific point in space, providing even more flexibility than earlier. On the right-hand side of the equation, we now deal with the exact exchange energy density, *∈*^ex^_x_, and a semi-local exchange energy density, *∈*^sl^_x_. The local hybrids offer a significant accuracy for the excitation energies, magnetic properties, and other basic molecular properties, while SIE is specially treated (removed or reduced) in different versions of the functional, providing more options depending on the systems in use. In our case we continue to find the optimal compromise for the description of level alignments and absorption spectra, hoping to have the same behaviour with the bifacial DSSCs.

## 3. Computational details

All calculations were performed with the TURBOMOLE program suit for the quantum-chemical simulations.^31^ The structures in this work were built and visualized with VESTA and Avogadro.^32,33^ Optimization of the structures were done by B3LYP exchange–correlation functional, 6-31G* basis set (applied to all atoms in the system), and m3 grid size.^34,35^ The conductor-like screening model (COSMO) is used in all calculations with standard settings for the acetonitrile as a solvent, since it has been proven in our previous work as highly soluble.^36^ Accurate representation of the semiconductor nanoparticles is established through the (TiO_2_)_38_ anatase cluster used in the previous studies.^37,38^ Mono- and bi-facial DSSC structures are treated at the same level of theory, allowing us to acquire more accurate results for the comparison. For the excited state calculations, addition to the B3LYP, we used the range-separated functional CAM-B3LYP and the local hybrid functional Lh12ct-SsifPW92 in order to test functional dependencies.^39–41^

## 4. Results

**Fig. 1:**
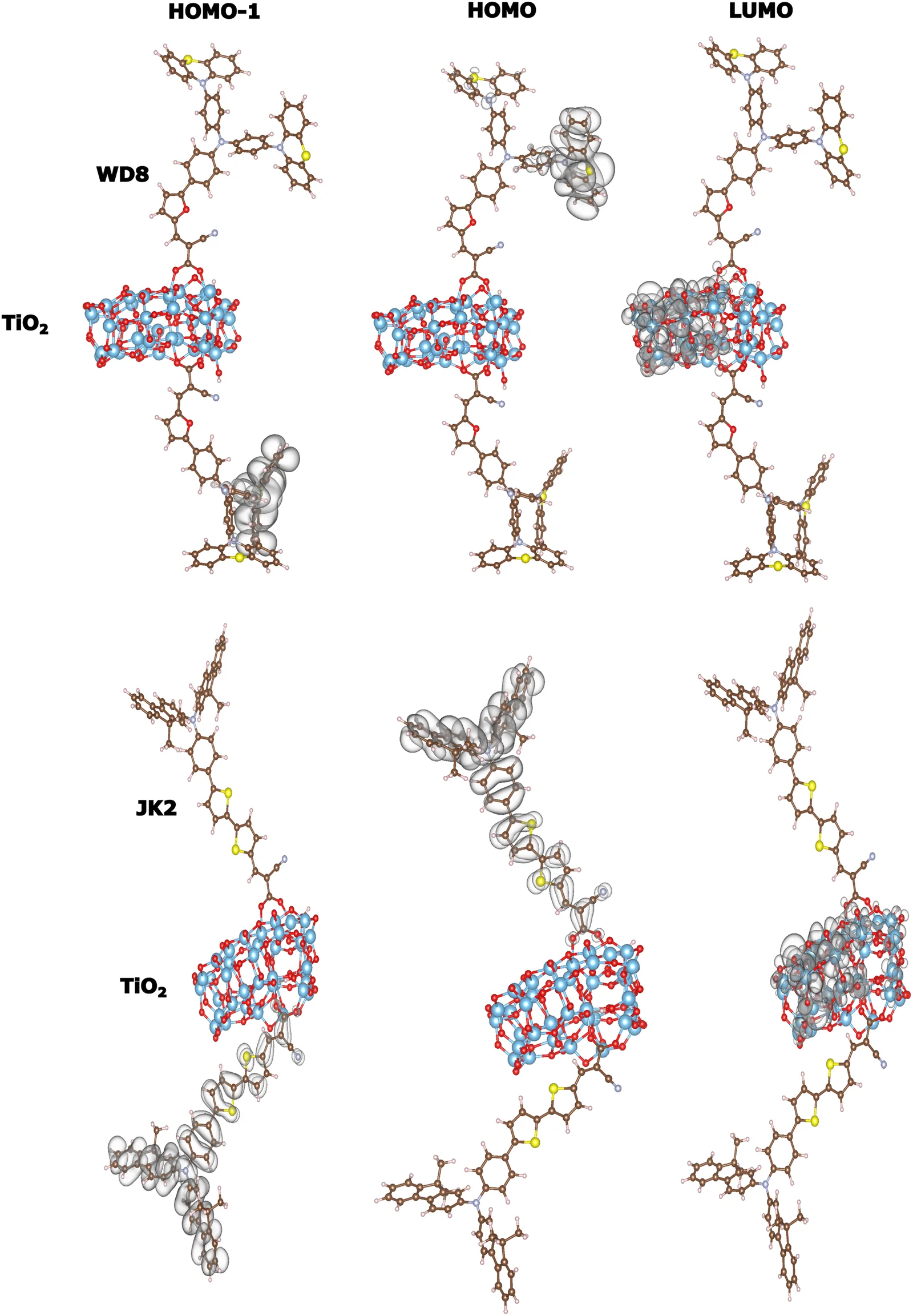
HOMO, HOMO−1, and LUMO charge distributions for the WD8 and JK2 bifacial DSSCs. The isocontour value is chosen as 0.01*a*_0_^−3^. Ti-atoms (blue), C-atoms (brown), N-atoms (grey), S-atoms (yellow), H-atoms (white), O-atoms (red).

**Fig. 2:**
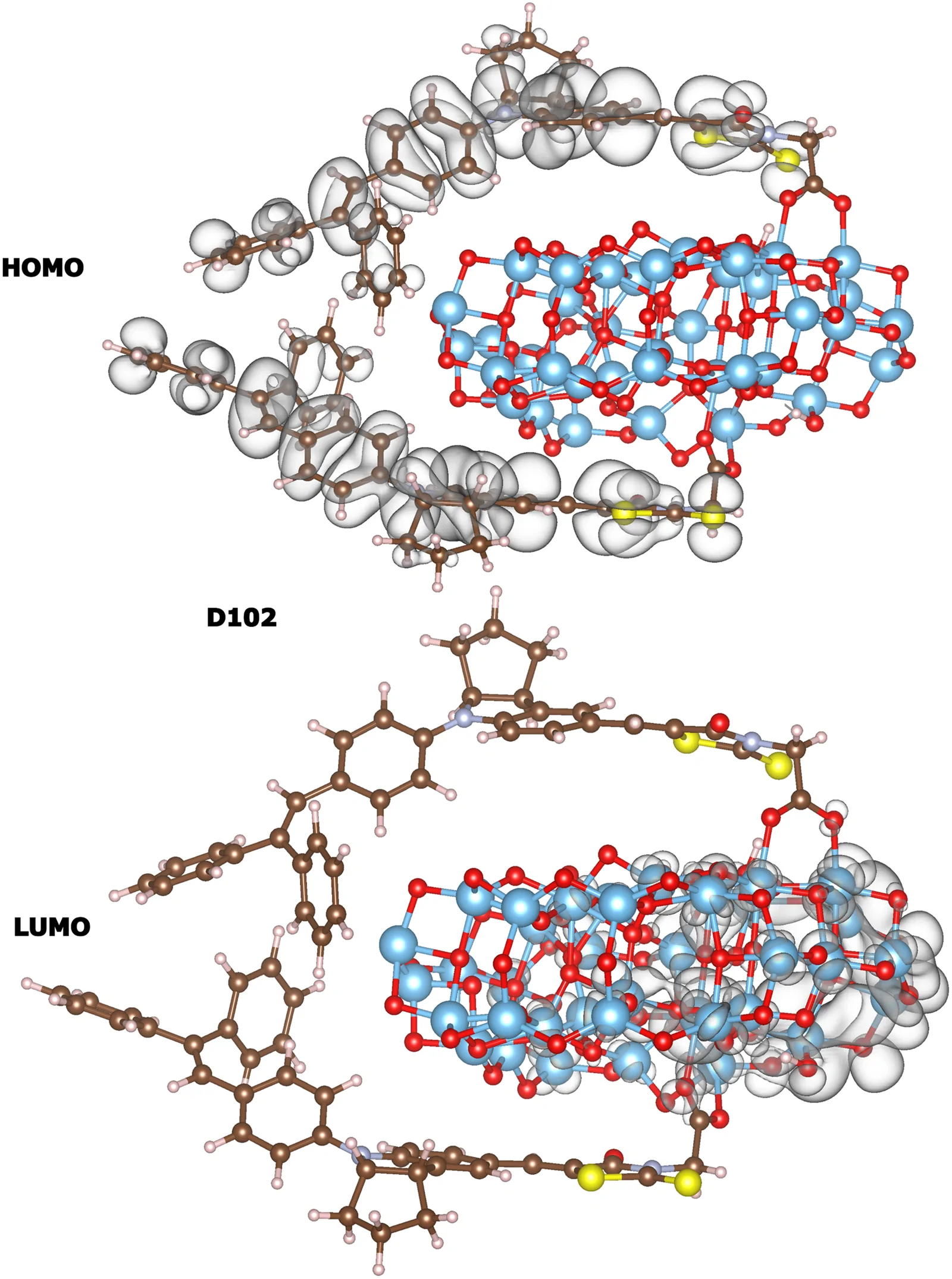
HOMO and LUMO charge distributions for the D102 bifacial DSSCs. The isocontour value is chosen as 0.01*a*_0_^−3^. Ti-atoms (blue), C-atoms (brown), N-atoms (grey), S-atoms (yellow), H-atoms (white), O-atoms (red).

**Fig. 3:**
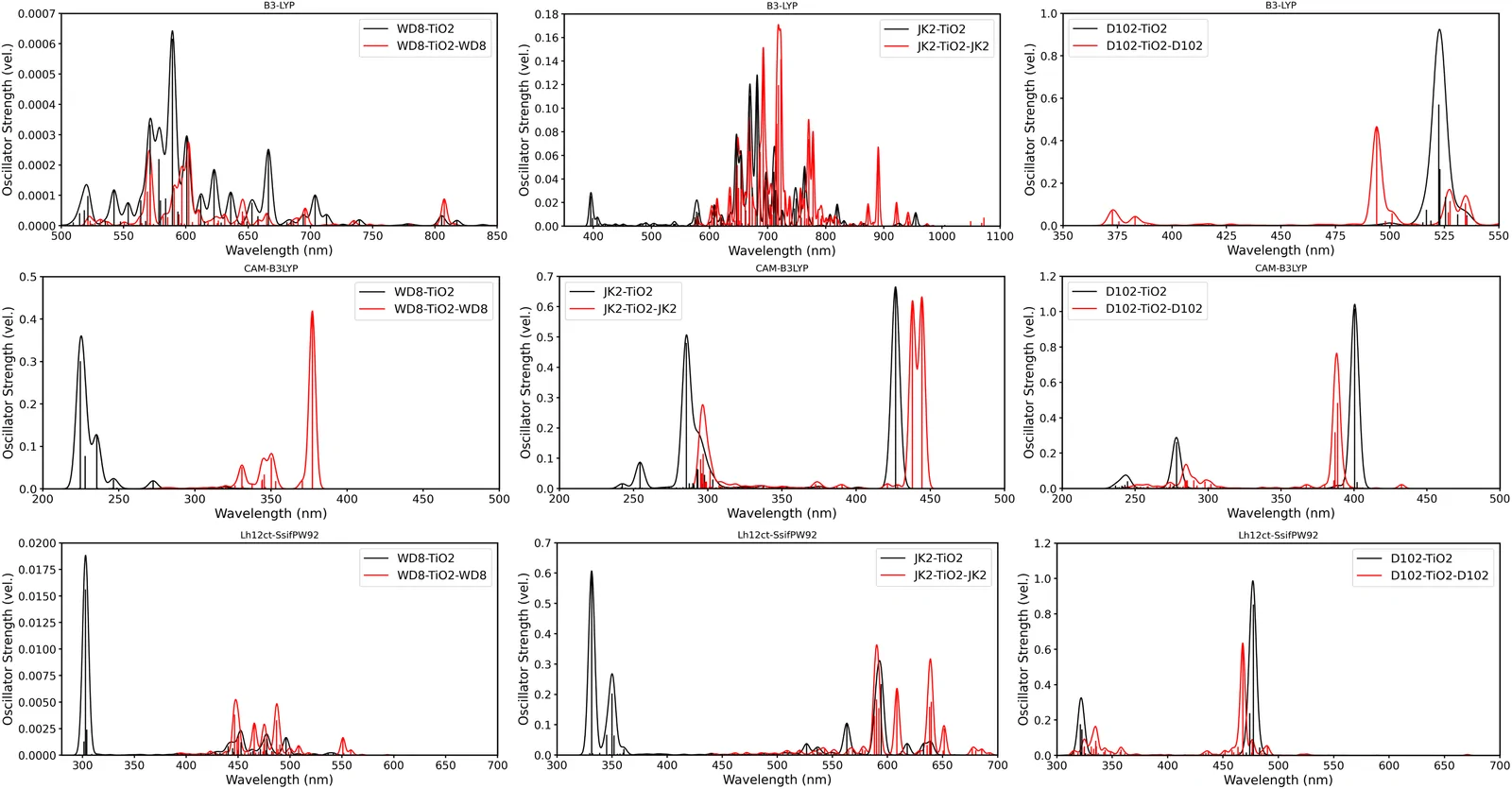
Absorption spectra broadened with a Gaussian distribution for the WD8, JK2 and D102 mono- and bi-facial systems with three different exchange correlation functionals: B3LYP, CAM-B3LYP, and Lh12ct-SsifPW92.

The first step in this work is to define the electronic structure and density redistribution in all three cases in the bifacial systems. With the monofacial counterpart, we already analyzed and concluded that the highest occupied molecular orbital (HOMO) is always located on the dye, while the lowest unoccupied molecular orbital (LUMO) is completely localized on the semiconductor. For the bifacial structures it is not that trivial, so it is of the highest importance to understand their frontier orbitals. Thus, Fig. 1 shows the WD8-TiO_2_-WD8 and JK2-TiO_2_-JK2 electronic density distribution. These two dyes exhibit an identical behavior where HOMO and HOMO−1 are localized at the front and the rear sides of the dye (DSSC), while LUMO is strictly on the TiO_2_. This suggests a weaker inter-dye coupling and it possibly leads to the two independent excitations. For the comparison, Fig. 2 shows a different situation of the charge distribution for the D102 dye, here HOMO is delocalized on the both sides of the dye, which could indicate strong coupling of states and potential enhancement of the charge transport. The differences can also be observed if we investigate absorption spectra for each structure with a direct comparison with the monofacial DSSCs, with three density functional approximations (Fig. 3). For the WD8 dye we can see how B3LYP is showing relatively weak charge transfer with very low oscillator strengths in both systems. While monofacial system shows few single peaks between 550 nm and 700 nm, bifacial is fragmented in few smaller peaks with infinitesimal red-shift. The latter can be explained with the weak interaction between the two dyes on both sides of the TiO_2_. Range separated functional CAM-B3LYP shows something very interesting – monofacial system has a single peak around 225 nm which is a natural π–π* transition, while bifacial system exhibits a dominant peak around 375 nm with increase in the oscillator strength. Here, we observe a new peak that has emerged as a new optical active mode. Bifacial WD8 system creates new intense absorption transitions which the monofacial system does not have. Reason for this lies in the combination of the HOMO–LUMO and HOMO−1–LUMO transition from both dyes. Local hybrid functional shows one strong peak for the monofacial system around 300 nm, while bifacial system has a group of peaks between 440 nm and 500 nm and a movement to the visible spectra. The Lh12ct-SsifPW92 functional only confirms what CAM-B3LYP showed us, giving new allowed transitions and the proof of how two WD8 dyes are connected *via* semiconductor, even though HOMO/HOMO−1 are not delocalized. We can conclude from the example of the WD8 dye that the bifacial arrangements do not simply double the absorption, rather they introduce new electronic couplings that generate new optical transitions which are not present in the monofacial structures. Since JK2 has the same charge distribution of the HOMO and HOMO−1, detailed examination of the JK2 mono- and bi-facial absorption spectra leads to the identical conclusions as for the WD8. Moving to the delocalized D102 dye, we can see how B3LYP has a very narrow π–π* peak at 525 nm with strong oscillator strength for the monofacial system, while bifacial DSSC splits the main peak into two absorptions around 505 nm and 515 nm, still keeping the total oscillator strength relatively high and some additional peaks around 375 nm. The system does not behave as two isolated dyes and shows how both dyes can “see” each other, while bifacial system changes the photophysical properties. The CAM-B3LYP shows something similar for the monofacial D102 system, which is one dominant peak that is slightly shifted for the bifacial system with some new, smaller peaks. At the local hybrid level, there is a strong peak for the monofacial system around 490 nm, and that peak splits up at approximately 2–3 states in the bifacial counterpart, where a new peak is observed around 460 nm and total oscillator strength is distributed between these states. In conclusion, we can confirm how, with B3LYP, there is a more pronounced splitting and redistribution of the main visible transition, which is consistent with a strong exciton coupling between two dyes, while CAM-B3LYP shows only modest broadening and a small blue shift of the main band, indicating weaker effective coupling. The Lh12ct-SsifPW92 is in-between, showing an intermediate case between the first two.

**Fig. 4:**
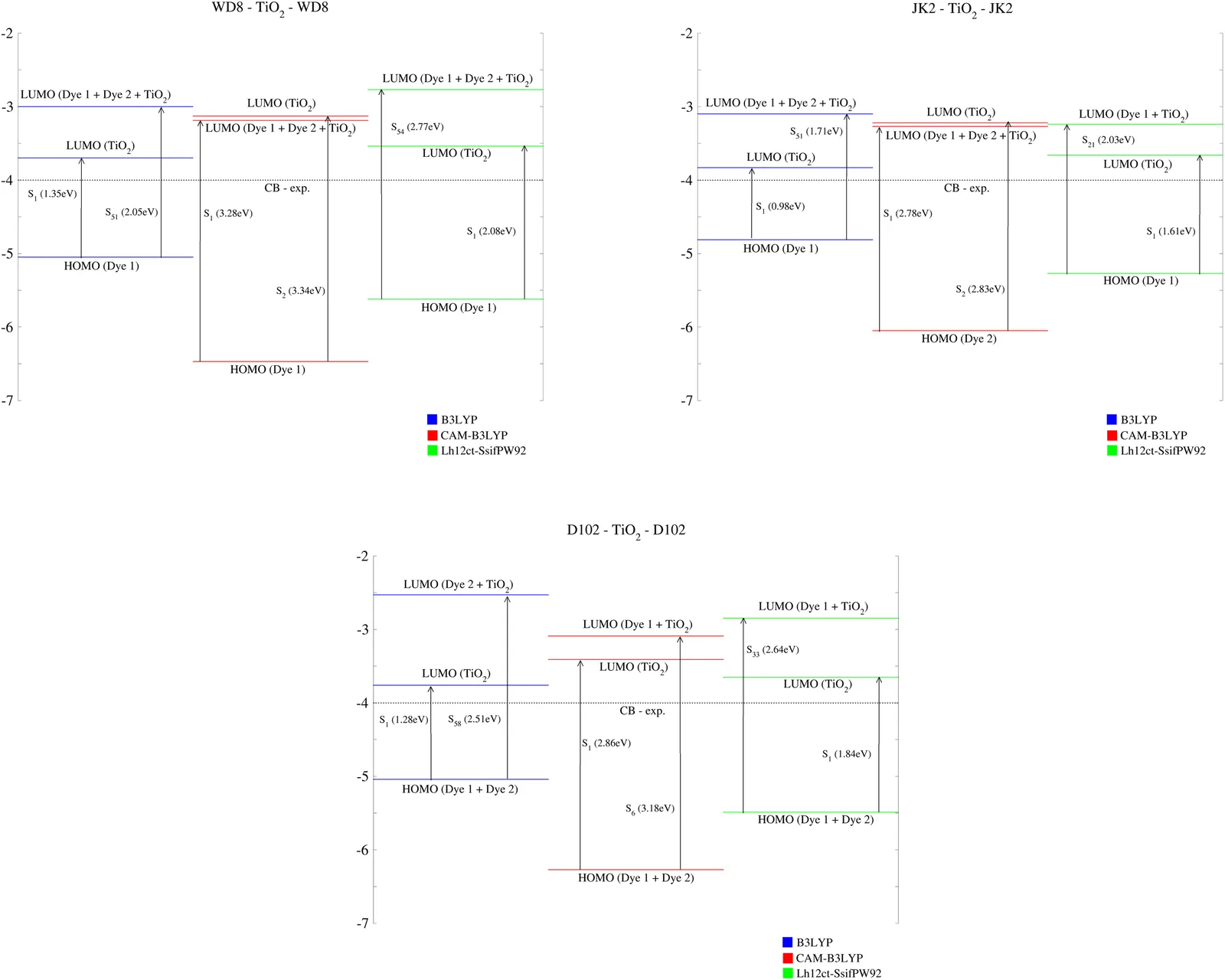
Alignment of the energy levels for the WD8, JK2, and D102 bifacial systems compared with three different exchange correlation functionals: B3LYP (blue), CAM-B3LYP (red), and Lh12ct-SsifPW92 (green). The black dotted line is representing the experimental TiO_2_ conduction band (CB). All vertical excitations from HOMO to LUMO are also represented together with the number of the excited state responsible for the respected excitation, S.

The energy level alignment obtained from the LR-TDDFT (Fig. 4) reveals a pronounced dependence on both the system (electronic structure) and the underlying exchange correlation functional. For all three bifacial systems (WD8, JK2, and D102) B3LYP and Lh12ct-SsifPW92 yield a consistent and physically reasonable alignment in which the dye frontier orbitals lie energetically above the TiO_2_ CB, thus supporting electron injection upon photoexcitation. On the other hand, range-separated functional CAM-B3LYP exhibits a markedly different behavior. For both WD8 and JK2 bifacial systems, we have a wrong level alignment at the interface characterized by a TiO_2_ LUMO lying below the dye LUMO, which leads to an inverted and nonphysical ordering of the levels, where the dye appears energetically lower than the TiO_2_. This difference can be traced back to the distinct orbital topology of the three bifacial systems. The D102 system features a delocalized HOMO on both dye moieties, resulting in a robust and functionally insensitive alignment, whereas WD8 and JK2 exhibit spatially separated HOMO and HOMO−1 orbitals localized on opposite dyes, allowing their electronic structure to be more sensitive to the long-range exchange treatment. These results also support our previously published findings, where we had a monofacial JK2-TiO_2_ and WD8-TiO_2_ systems which showed an identical behavior with the CAM-B3LYP, providing exactly the same level alignment prediction. It should be emphasized that the objective of this work is not to derive molecular design rules for new sensitizers, but rather to understand how representative dye structures behave upon transitioning from mono- to bifacial adsorption.

Table 1 shows calculated band gaps, (*E*_g_), obtained from the difference between HOMO and LUMO energies, light harvesting efficiencies, (LHE), and bifaciality factor, (BFF), with direct comparison between monofacial and bifacial systems and their functional dependencies. As in our previous work, to calculate LHEs of the systems we used Beer's law:

4 LHE = 1 − 10^−*f*^.

where *f* is the oscillator strength linked to the corresponding maximum absorption peak. For the BFF we used both the front and the rear sides of the cell, and total oscillator strength which is the sum of *f* produced by HOMO and HOMO−1 orbitals. In the absence of an explicit illumination direction in first-principles calculations the notions of the front- and the rear-side excitation are defined operationally in terms of orbital localization. Excitations originating from frontier orbitals localized on opposite dye moieties are interpreted as front- and rear-side photoexcitation, respectively. The BFF is calculated as:5
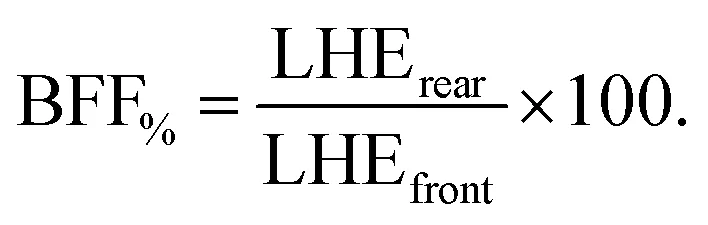


**Table 1:** Calculated band gaps, (*E*_g_), light harvesting efficiencies, (LHEs), and the bifaciality factor, (BFF), for each system and functional

Functional	System	*E*_g_ [eV]	LHE	BFF%
B3LYP	D102-TiO_2_	1.52	0.73	—
	D102-TiO_2_-D102	1.52	0.65	—
	JK2-TiO_2_	1.15	0.25	—
	JK2-TiO_2_-JK2	1.17	0.55	98
	WD8-TiO_2_	1.37	0.00076	—
	WD8-TiO_2_-WD8	1.49	0.00099	63.33
CAM-B3LYP	D102-TiO_2_	3.89	0.90	—
	D102-TiO_2_-D102	3.89	0.67	—
	JK2-TiO_2_	3.53	0.78	—
	JK2-TiO_2_-JK2	3.52	0.99	29.87
	WD8-TiO_2_	3.96	0.49	—
	WD8-TiO_2_-WD8	4.01	0.77	24.76
Lh12ct-SsifPW92	D102-TiO_2_	2.26	0.86	—
	D102-TiO_2_-D102	2.25	0.76	—
	JK2-TiO_2_	1.88	0.74	—
	JK2-TiO_2_-JK2	1.90	0.73	87.51
	WD8-TiO_2_	2.21	0.035	—
	WD8-TiO_2_-WD8	2.31	0.0138	86.48

Evaluation of the BFF was done for the WD8 and JK2 systems only, while it was not reported for the D102. This choice is motivated by the distinct orbital topology, as we already established. For the D102 system, HOMO is delocalized over both dyes, resulting in a single, electronically symmetric donor state. Thus, photoexcitation from either side of the bifacial interface leads to the equivalent electronic pathways, where BFF is trivial and approaches 100% by the sole construction without additional physical insight. In contrast, both WD8 and JK2 exhibit spatially separated HOMO and HOMO−1 orbitals, and the evaluation of these systems captures directional asymmetry and could be meaningful.

The data from Table 1 provide a comprehensive overview of distinct electronic trends, depending on both the employed systems and the used functionals. For the D102 systems, the calculated band gap remains unchanged when we move from the monofacial to the bifacial system across all functionals, where both dye units contribute symmetrically to the HOMO, resulting in an equal excitation landscape. For the WD8 and JK2, introduction of the second dye induces small changes, which originate again from the HOMO and HOMO−1 localized on opposite dyes. The obtained BFF values show their signatures throughout the different functionals. The B3LYP predicts a moderate bifacial asymmetry, CAM-B3LYP leads to strongly suppressed rear-side contributions, and this bahavior correlates directly with the treatment of spatially separated frontier orbitals, showing how excessive long-range exchange contribution may artificially enhance asymmetry. The local hybrid functional yields nearly symmetric bifacial response, as a consequence of its adaptive treatment of exact exchange, reflecting the most realistic scenario in which both sides of the bifacial architecture contribute efficiently to the light harvesting process.

## 5. Conclusion and outlook

In this work, we carried out a comprehensive first-principles investigation of bifacial dye@TiO_2_ interfaces to elucidate the electronic and optical effects of bifacial dye functionalization. The obtained results were compared with the monofacial dye@TiO_2_ systems reported in our previous work. Using a combination of DFT and LR-TDDFT calculations, three representative organic dyes (D102, JK2, and WD8) were systematically analyzed by employing a global hybrid, a range-separated hybrid, and a local hybrid functional. The results reveal that the electronic response of bifacial systems is governed primarily by the spatial topology of the frontier orbitals rather than by the mere presence of an additional dye molecule. For the D102, the bifacial configuration preserves an essentially identical electronic structure and band gap compared to the monofacial system across all functionals, reflecting the delocalized nature of the HOMO over both dye units. In this case, the bifacial response is intrinsically symmetric, rendering directional metrics such as the bifaciality factor physically trivial. In contrast, JK2 and WD8 exhibit spatially separated HOMO and HOMO−1 orbitals localized on opposite dye units, enabling distinct excitation pathways associated with front- and rear-side illumination. This orbital separation leads to subtle band gap modifications upon bifacial functionalization and allows for a meaningful quantification of bifaciality. The computed bifaciality factors for the JK2 and WD8 display a pronounced dependence on the choice of exchange–correlation functional. While the B3LYP predicts a moderate asymmetry and the CAM-B3LYP leads to strongly suppressed rear-side contributions due to an overemphasis of long-range exchange effects, the local hybrid functional yields a nearly symmetric yet non-trivial bifacial response. This behavior correlates directly with its ability to preserve orbital continuity while accounting for spatial variations in exchange, suggesting that local hybrid approaches provide the most physically consistent description of bifacial dye@TiO_2_ interfaces. Overall, this study demonstrates that bifaciality in dye-sensitized architectures is not an inherent property but emerges from the interplay between dye orbital topology and the theoretical treatment of electronic exchange. By establishing a clear structure–property relationships and highlighting the critical role of functional choice, the presented work provides a robust theoretical framework for the rational design and interpretation of the bifacial dye-sensitized systems, offering valuable guidance for future experimental and computational developments.

## Author contributions

All authors contributed to this work.

## Conflicts of interest

There are no conflicts to declare.

## Data Availability

The data that support the findings of this study are available from the corresponding author upon reasonable request.
